# Delayed Functional Networks Development and Altered Fast Oscillation Dynamics in a Rat Model of Cortical Malformation

**DOI:** 10.3389/fnins.2020.00711

**Published:** 2020-08-18

**Authors:** Min-Jee Kim, Mi-Sun Yum, Youngheun Jo, Minyoung Lee, Eun-Jin Kim, Woo-Hyun Shim, Tae-Sung Ko

**Affiliations:** ^1^Department of Pediatrics, Asan Medical Center Children’s Hospital, University of Ulsan College of Medicine, Seoul, South Korea; ^2^Department of Radiology, Asan Medical Center, University of Ulsan College of Medicine, Seoul, South Korea

**Keywords:** malformations of cortical development, resting state functional magnetic resonance images, functional connectivity, event-related spectral perturbation, gamma, ripples

## Abstract

Malformations of cortical development (MCD) is associated with a wide range of developmental delay and drug resistant epilepsy in children. By using resting-state functional magnetic resonance imaging (RS-fMRI) and event-related spectral perturbation (ERSP) of cortical electroencephalography (EEG) data, we tried to investigate the neural changes of spatiotemporal functional connectivity (FC) and fast oscillation (FO) dynamics in a rat model of methylazoxymethanol (MAM)-induced MCD. A total of 28 infant rats with prenatal exposure to MAM and those of age matched 28 controls with prenatal saline exposure were used. RS-fMRI were acquired at postnatal day 15 (P15) and 29 (P29), and correlation coefficient analysis of eleven region of interests (ROI) was done to find the differences of functional networks between four groups. Two hour-cortical EEGs were also recorded at P15 and P29 and the ERSP of gamma (30–80 Hz) and ripples (80–200 Hz) were analyzed. The rats with MCD showed significantly delayed development of superior colliculus-brainstem network compared to control rats at P15. In contrast to marked maturation of default mode network (DMN) in controls from P15 to P29, there was no clear development in MCD rats. The MCD rats showed significantly higher cortical gamma and ripples-ERSP at P15 and lower cortical ripples-ERSP at P29 than those of control rats. This study demonstrated delayed development of FC and altered cortical FO dynamics in rats with malformed brain. The results should be further investigated in terms of the epileptogenesis and cognitive dysfunction in patients with MCD.

## Introduction

Malformations of cortical development (MCD) is a group of disorders that has disruption at any step of human brain development including cell proliferation, neuronal migration, or post-migrational cortical organization and connectivity (Barkovich et al., 2012; Guerrini and Dobyns, 2014). The clinical feature and course of MCD is widely varied from normal to severe cognitive impairment, and epilepsy is one of the most common presenting symptoms. Moreover, near 90% of patients with epilepsy caused by MCD has been reported to be drug-resistant (Guerrini and Dobyns, 2014).

Although there has rapid evolution of molecular biology, genetics, and neuroimaging techniques in clinics, studies with animal models are still needed for the investigation of MCD pathogenesis due to limitation of clinical studies involving patients or human tissues. There are several animal models of MCD (Luhmann, 2016) and one such model is the prenatal methylazoxymethanol (MAM)-induced MCD model. Prenatal exposure of MAM disrupts cell migration resulting the malformations mostly in the hippocampal CA1 and CA2 region, to a lesser extent in the striatum, thalamus, hypothalamus, and cerebral cortex, which are similar to MCDs in human patients (Spatz and Laqueur, 1968; Singh, 1977; Luhmann, 2016). Spontaneous epileptic seizures have not been reported so far but several studies indicated a lower threshold for seizures or epileptiform activities (Baraban and Schwartzkroin, 1995; Colciaghi et al., 2011; Kim et al., 2017). MAM-treated rats also had behavioral alteration even at infancy which is consistent with cognitive impairment in patients with MCD (Lucas et al., 2011; Kim et al., 2017).

Recently, resting-state functional magnetic resonance imaging (RS-fMRI) is widely used for mapping large-scale brain networks, the intrinsic functional connectivity (FC) of brain without external stimuli in humans and animal models (Fox et al., 2005; Raichle and Mintun, 2006). Although RS-fMRI on small animals is still scarce, they have the potential to identify the pathologic brain circuits responsible for a neurological disorder. We hypothesized that the structural brain malformation caused the disrupted FC which is associated with behavioral alteration and susceptibility to seizures in MCD rats, and tried to find the abnormal FC of MCD rats using RS-fMRI.

Unfortunately, RS-fMRI is indirect and a low time resolution method to investigate local neuronal activities associated with malformed cortex. As electroencephalography (EEG) is another measure of complex structural-functional dynamics which reflects a direct neuronal activity in high temporal resolution, the interest of combining these two modalities, RS-fMRI and EEG, has grown markedly in last two decades (Sumiyoshi et al., 2011; Wirsich et al., 2017). Classically, cortical EEG has been used to find the epileptic networks or seizure onset zone in human and animal models. High frequency oscillations (HFOs) including gamma oscillation has been suggested as a marker for physiologic phenomenon of cognition (Nowak et al., 2018) or pathologic seizure onset zone recently (Jobst and Engel, 2015). To quantify the changes of these HFO, several measurement indices are used and event-related spectral perturbation (ERSP) was one of them (Delorme and Makeig, 2004; Makeig et al., 2004). Previously, our group also showed the elevated fast oscillation-event-related spectral perturbations (FO-ERSPs) of the pathologic brain lesions in patients with hypsarrhythmia (Kim et al., 2018).

To understand the mechanism of epileptogenicity and cognitive impairment of the brain with MCD, prenatally MAM-treated rats were used in this study. It is hypothesized that prenatally MAM-exposed MCD rats show disrupted resting state FC and abnormal FO dynamics during their brain development. To test this hypothesis and quantitatively measure the changes of the neural system, the FC maps of RS-fMRI data and the cortical FO dynamics were compared between rats with MCD and controls at their age of P15 and P29.

## Materials and Methods

### Animals

All experiments were approved by the by the Institutional Animal Care and Use Committee of the University of Ulsan College of Medicine and conducted in accordance with the Revised Guide for the Care and Use of Laboratory Animals. Pregnant Sprague–Dawley rat dams (*n* = 10, Orient Bio Inc., Seoul, South Korea) were acquired on gestational day (G) 14 and housed individually in the animal facility during the remainder of their pregnancy under a 12-h light/dark cycle with free access to food and water. They were injected intraperitoneally with either 0.9% physiological saline (control group, *n* = 5) or two doses of MAM (15 mg/kg/dose, MRIGlobal, Kansas City, MO, United States) in 10 mL/kg saline at 0800 and 1800 (MCD group, *n* = 5) on G15. Delivery occurred consistently on G22, which was considered postnatal day (P) 0 for the offspring. The rats used in these experiments are listed in Supplementary Table S1.

### Magnetic Resonance Imaging (MRI) Acquisition

Each of eight MCD and control rats was used for imaging data at P15 and P29. All MRI studies were conducted using a 7.0 T/160-mm small-animal imaging system (Bruker Pharmascan, Ettlingen, Germany) with 400 mT/m gradient system and a surface coil for reception and 72 mm volume coil for transmission. During scanning, rats were anesthetized with mixture of 1.0% isoflurane and room air delivered by nose cone and their respiratory rate, electrocardiogram, and the rectal temperature were monitored using small-animal physiological monitoring devices. High-resolution anatomical T2 weighted images were acquired with rapid acquisition with relaxation enhancement (RARE) sequence [TR/TE = 4000/33.0 ms, rare factor = 8, slice thickness = 0.8 mm (total 20 slices), matrix size = 256 × 256, and field of view (FOV) = 25 × 25 mm]. Functional MR data were acquired using a single shot gradient echo based echo-planar image (GE-EPI) sequence positioned parallel to the anterior–posterior commissure plane over the entire brain (TR/TE = 1000/16.734 ms, flip angle = 35′, 300 repetitions, FOV = 25 × 25 mm, matrix size = 96 × 96, no inter slice gap with 10 axial slices).

### Thickness and Size Measurement of Neocortex, Hippocampus, and Ventricle

**FIGURE 1:**
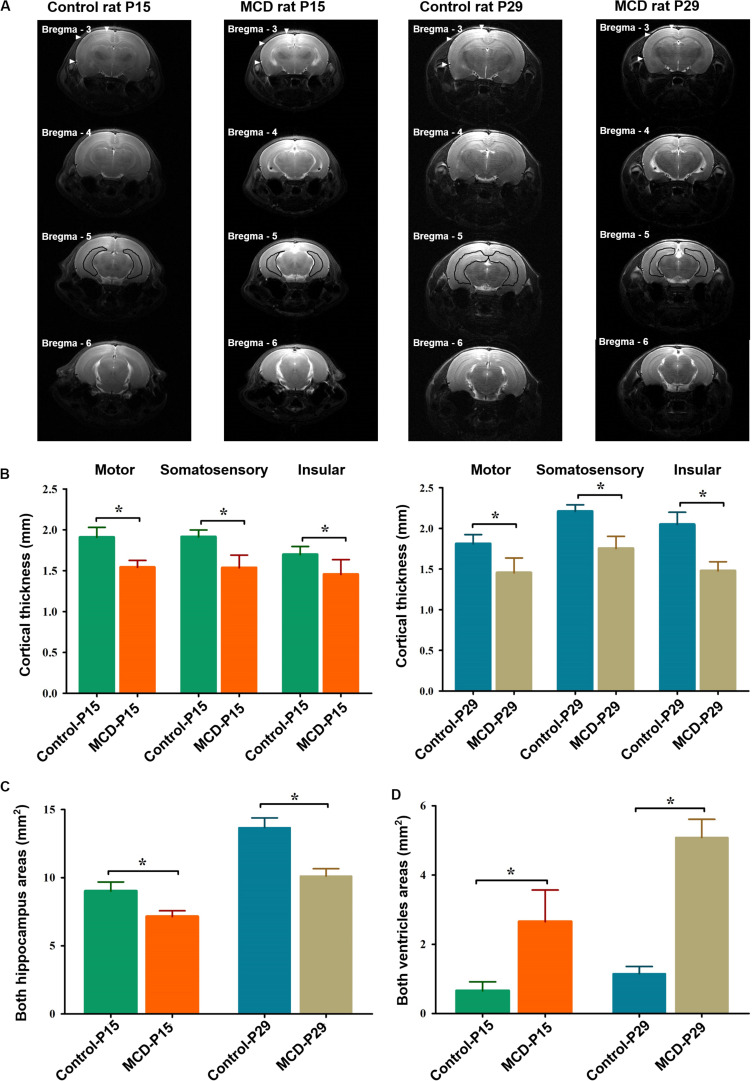
Representative high-resolution anatomical images and comparisons of cortical thickness, hippocampal, and ventricle sizes in each group. **(A)** The depth of the motor, somatosensory, and insular cortices [filled arrowheads at bregma, −3 mm AP (anterior to posterior)], hippocampal (black lines at bregma -5 mm AP), and ventricle areas (black asterisks at bregma, −4 mm AP) were measured in control and malformations of cortical development (MCD) rats at postnatal day 15 (P15) and 29 (P29). Compared to controls, MCD rats showed significant **(B)** thinning of motor (P15; 1.54 ± 0.08 mm vs. 1.91 ± 0.12 mm, P29; 1.45 ± 0.18 vs. 1.81 ± 0.11 mm, unpaired *t*-test, *p* = 0.001), somatosensory (P15: 1.54 ± 0.08 mm vs. 1.91 ± 0.12 mm; P29: 1.45 ± 0.18 vs. 1.81 ± 0.11 mm, unpaired *t*-test, *p* = 0.001), and insular (P15: 1.45 ± 0.18 mm vs. 1.70 ± 0.10 mm; P29: 1.48 ± 0.11 mm vs. 2.05 ± 0.15 mm, unpaired *t*-test, *p* = 0.001) cortices, **(C)** area reduction in both hippocampi (P15: 7.14 ± 0.43 mm vs. 9.02 ± 0.67 mm; P29: 10.11 ± 0.54 mm vs. 13.67 ± 0.71 mm, paired *t*-test, *p* = 0.001) and **(D)** enlarged ventricles (P15: 2.65 ± 0.91 mm vs. 0.66 ± 0.26 mm; P29: 5.08 ± 0.52 mm vs. 1.15 ± 0.21 mm, unpaired *t*-test, *p* = 0.001) at both developmental ages. *p < 0.05.

To assess the morphological changes of MCD rats at different developmental ages, the neocortical depths, hippocampal, and ventricle size were analyzed with high resolution T2 weighted images using SPM12 and ImageJ^1^. To measure the same cortical point of each rat, the anatomical images at of all subjects were overlaid and co-registered each other using SPM12. The cortical thickness of motor, somatosensory, and insular cortices in 3 mm posterior from the bregma, the areas of both hippocampi at 5 mm posterior from the bregma, and the lateral ventricles at 4 mm posterior from the bregma were measured (Figure 1A).

### Resting-State Networks Analysis

#### Preprocessing

Several steps of preprocessing were performed using MRIcron software^2^ and AFNI software (Analysis of Functional NeuroImages^3^). Preprocessing steps included: (i) *Brain extraction:* Brain extraction was done manually using MRIcron software. (ii) *Registration to standard space:* The registrations of functional image to anatomical data and anatomical data to a high-resolution rat brain template were carried out using (FMRIB software library) FSL’s flirt (FMRIB’s Linear Image Registration Tool^4^) using 12° of freedom affine transformation. The rat brain template was selected for a T2 weighted anatomical scan with field of view of 136 mm × 102 mm × 71 mm and spatial resolution of 1.25 mm × 1.25 mm × 1.25 mm mm (Scalable Brain Atlas^5^). (iii) *Data cleanup:* Discarding the first three volumes, slice timing correction, detrending, despiking, motion correction, ventricular, and global signal regression. (iv) *Band-pass filtering:* Functional images were band-pass filtered between 0.01 and 0.1 Hz. (v) *Spatial smoothing:* We applied the spatial smoothing using Gaussian kernel FWHM of 0.6 mm to identify relatively large-scale networks across the whole brain of a young rat.

#### Network Analysis

Resting state analysis was performed using spatial ICA (independent component analysis) by the tool MELODIC (Multivariate Exploratory Linear Optimized Decomposition into Independent Components) from FSL. ICA is being successfully applied method in diverse range of neuroscience for automatically separating various “independent” sources (James and Hesse, 2005; Gorges et al., 2017). To find the common spatial patterns of each four groups, we used group-ICA (Calhoun et al., 2001; Beckmann and Smith, 2004) on the entire set of fMRI data concatenated across the subject of each group. The extracting components were set to 40, which seem to be a reasonable number to provide sub-network separation without incurring into mathematical granularity that produces individual structures and components (Hutchison et al., 2010; Liang et al., 2011).

To assess FC change of the individual part with structural abnormalities, five neocortical areas [primary/secondary visual cortex/posterior parietal cortices (V1/V2/PPC), somatosensory cortex (SS), motor cortex (Motor), auditory cortex (Au), and anterior/posterior cingulate cortices (aCg/pCg)], five subcortical areas [hippocampus, basal ganglia, thalamus, hypothalamus, and superior colliculus (SC)] and brainstem were designated the region of interests (ROI) and the Pearson correlation coefficient (CC) among the mean signal intensity time courses of total 11 ROI were calculated. We represent the 11 ROI in Supplementary Figure S1. There was a large gap in the brain size and morphology between P15 and P29, we used the different masks for those P15 and P29 rats. A Fisher’s r-to-z transformation was applied to each correlation map to obtain an approximately normal distribution of the FC values. Two CC maps were compared between those with rats with MCD and controls at each timepoint P15 and P29 to show the abnormal FC of MCD rats during early developmental period. To adjust the random effect of clustering from the same mother and repeated measure effect at two timepoints, we performed the linear mixed model analysis.

### Cortical Electroencephalography (EEG) Recording and Analysis

For intracranial EEG recording, electrodes were surgically implanted in each of ten MCD and control rats under sedation with ketamine/xylazine (50/7 mg/kg in 10 mL/kg saline IP) at P13 and P27, respectively. Two cortical electrodes were implanted over both somatosensory cortices in each of five MCD and control rats. They are connected to a multiple socket and secured to the skull with dental acrylic. At P15 and P29, 2-h non-sedated EEGs of cortical electrodes were recorded with simultaneous and synchronized video using the Twin EEG system (Grass Technologies Corp.). The sampling rate was 400 Hz with 0.1 Hz high-pass filter. The 80 s of each artifact-free, resting state EEG were collected and reassessed. The extracted EEGs were extended temporally with 1, 30, and 80 Hz high pass filters to detect visible gamma (30–80 Hz) and ripples (80–200 Hz) with sensitivities of 50 and 25 μV/mm, respectively. For quantitative estimation, ERSP of gamma and ripples from each epoch was calculated using the EEGLAB toolbox of MATLAB 2017b. ERSP (log) was used to show dynamic brain changes, with the zero point in each epoch set as the baseline. In each 1-s epoch, ERSPs were analyzed with fast Fourier transform and Hanning window tapering. The ERSP formula for averaged estimates across data trials (*n* trials) is defined below:

$$\mathrm{ERSP}\,\left(f,t\right)=\frac{1}{n}\,\sum_{k=1}^{n}{\left|F_{k}\,\left(f,t\right)\right|}^{2}$$

where *F*_*k*_(*f,t*) is the spectral estimate of trial *k* at frequency *f* and time *t*.

The cortical gamma and ripples-ERSP extracted from 80 epoch in each rat were compared between those with MCD rats and controls at different ages, P15 and P29 using linear mixed model analysis with adjusting random effects of clustering from same mother and repetitive measured values (80 epoch) from each rat.

## Results

### Comparisons of Cortical Thickness, Hippocampal, and Ventricle Sizes

Using *in vivo* MRI, we could demonstrate the widespread cortical thinning of MCD rats of this study in motor, somatosensory and insular cortices when compared to control rats (Figure 1, unpaired *t*-test, P15-motor: *t* = 7.001, df = 12.507, *p* = 0.001; P15-somatosensory: *t* = 6.032, df = 10.761, *p* = 0.001; P15-insular: *t* = 3.348, df = 10.864, *p* = 0.007; P29-motor: *t* = 4.703, df = 11.703, *p* = 0.001; P29-somatosensory: *t* = 7.605, df = 10.748, *p* = 0.001; P29-insular: *t* = 8.576, df = 12.916, *p* = 0.001) as previously shown (Kim et al., 2017). The two-dimensional measurements of bilateral hippocampi and lateral ventricles also revealed significant reduction in those of MCD rats at P15 and P29 (Unpaired *t*-test; P15-hippo: *t* = 6.632, df = 11.854, *p* = 0.001; P15-ventricle: *t* = 5.939, df = 8.115, *p* = 0.001; P29-hippo: *t* = 11.187, df = 13.060, *p* = 0.001; P29-ventricle: *t* = 19.702, df = 9.175, *p* = 0.001).

### Resting-State Networks Identification

**FIGURE 2:**
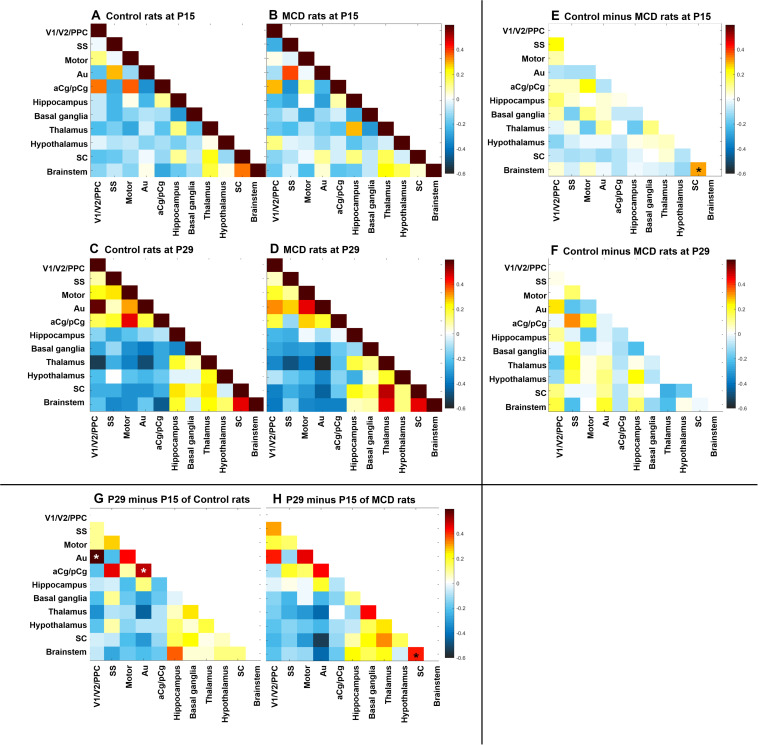
Correlation coefficient map of functional connectivity (FC). **(A–D)** Correlation coefficient matrices representing FC among the 11 region of interests (ROI) of control rats at P15 **(A)**, P29 **(C)** and rats with MCD at P15 **(B)**, P29 **(D)**. **(E,F)** Subtraction maps presenting the correlation coefficient differences between MCD and control rats at P15 **(E)** and P29 **(F)**. **(G,H)** Subtraction maps of two different age, P15 and P29 of controls **(G)** and MCD rats **(H)** *Linear mixed model analysis, *p < 0.05.

**FIGURE 3:**
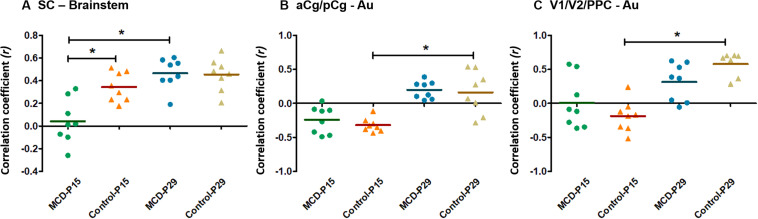
Scatter dot plots of correlation coefficient (CC) in controls and malformations of cortical development (MCD) rats at P15 and P29. Green circle: MCD rats at P15, Orange triangle: Control rats at P15, Blue circle: MCD rats at P29, Yellow triangle: Control rats at P29. **(A)** CC between superior colliculus (SC) and brainstem; there were significant decrease of CC between SC and brainstem in MCD rats at P15 compared to controls and significant increase of CC between those from P15 to P29. **(B)** CC between anterior (aCg)/posterior cingulate cortex (pCg) and auditory cortex (Au); there were significantly increased in CC between aCg/pCg and Au in control group during developmental periods. **(C)** CC between primary (V1)/secondary visual cortex (V2)/posterior parietal cortex (PPC) and auditory cortex (Au); there were significantly increased in CC between V1/V2/PPC and Au in control group during developmental periods. ^∗^Linear mixed model analysis, ^∗^*p* < 0.05.

Using group-ICA, four functional networks including default mode, motor-somatosensory, basal ganglia-hypothalamus-hippocampus, and brainstem network were found in each group (Supplementary Figure S2). To quantitatively measure the group difference of FC between the cortical and subcortical areas, the correlation coefficient (CC) among 11 ROIs were analyzed and compared to each other. At P15, the whole brain FC was lower in both groups (Figures 2A,B) and the intra-cortical, intra-subcortical positive FC and neocortical-subcortical negative connectivity became stronger in both groups at P29 (Figures 2C,D). The MCD rats showed significantly reduced SC-brainstem connectivity compared to control rats at P15 and this difference disappeared at P29 (Figures 2E,F; P15: *t* = 4.45, *p* = 0.047; P29: *t* = −0.03, *p* = 0.976). When we compare the FC of P15 and P29 in each group, control rats showed a significant increase in aCg/pCg-Au FC (Figure 2G, *t* = −4.97, *p* = 0.038) and V1/V2/PPC-Au FC (Figure 2G, *t* = −6.13, *p* = 0.026) and MCD rats showed a significant increase in SC-brainstem FC (Figure 2H, *t* = −5.4, *p* = 0.033) from P15 to P29. To show the effect magnitude of cortical malformation and development, the CC of these significantly changed networks were shown in Figure 3.

### Cortical EEG Analysis

**FIGURE 4:**
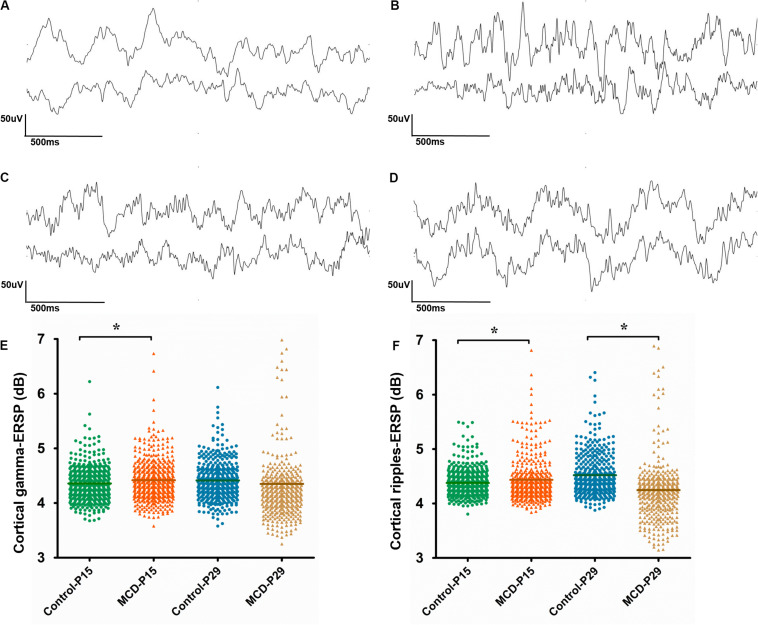
Analysis of cortical and hippocampal electroencephalography (EEG). **(A–D)** Example of visual analysis of raw EEG data with a 1 Hz high pass filter and a sensitivity of 50 μV/mm from control group aged P15 **(A)**, rats with malformations of cortical development (MCD) aged P15 **(B)**, control group aged P29 **(C)**, and rats with MCD aged P29 **(D)**. **(E,F)** Scatter dot plots of the cortical gamma **(E)** and cortical ripple **(F)** event-related spectral perturbations (ESRPs). First (green circle) and second (orange triangle) clusters of dots show control and rats with MCD at P15, respectively; the mean cortical gamma and ripples-ERSPs of rats with MCD were significantly higher than control group. Third (blue circle) and fourth (yellow triangle) clusters of dots show controls and rats with MCD at P29, respectively; the mean cortical ripples-ERSPs of rat with MCD were significantly lower than that of control group. *Linear mixed model analysis, *p < 0.05.

With 1 Hz high pass filters, visual analysis of cortical EEG showed relatively increase of fast activities in MCD rats especially at P15. The examples of EEGs on visual analysis are shown in Figure 4.

To quantify the time-related shift of FO, the mean cortical gamma and ripples-ERSP of rats with MCD or controls were compared at P15 and P29. The MCD rats at P15 showed significantly higher gamma- and ripple-ERSPs than controls (Figures 4E,F, cortical gamma: 4.41 ± 0.40 vs. 4.36 ± 0.32, *p* = 0.021, df = 278.906; cortical ripples: 4.43 ± 0.53 vs. 4.38 ± 0.24, *p* = 0.029, df = 201.113). Whereas cortical ripples-ERSP of MCD rats at P29 was significantly lower than that of control rats (Figure 4F, 4.25 ± 0.61 vs. 4.52 ± 0.39; *p* < 0.001; df = 119.808).

## Discussion

Malformations of cortical development are commonly associated with refractory epilepsy and cognitive impairment in children, yet the brain network alteration and developmental electrophysiological changes of this structurally abnormal brain are not clearly defined. Using a rat model of MCD, we analyzed the resting-state FC and FO dynamics of MCD brains to identify the developmental changes of functional networks and cortical electrical activities caused by the malformed brain in this study.

Both in humans and animal models, RS-fMRI has been used to study FC during rest (Smith et al., 2013; Smitha et al., 2017; Mandino et al., 2019) and this spontaneous fluctuation of BOLD signal was seen as similar pattern within same species (e.g., rats) or different species such as human and rodents (Sierakowiak et al., 2015). In this study, intrinsic connectivity networks (ICN) were successfully identified in all four groups (Supplementary Figure S2) which was consistent with the previously established essential networks in the anesthetized (Hutchison et al., 2010; Lu et al., 2012) or awake adult rats (Becerra et al., 2011), and 2-week-old infant rats (Bajic et al., 2016). Furthermore, this study also demonstrated the serial representative FC pattern at two different developmental ages.

To gain insight for the impaired network properties in diseased brain, it is necessary to examine whether there is significant difference in the degree of variability between the control and disease group (Hutchison et al., 2013; Hillary et al., 2015). At first, we could confirm the widespread structural disruptions in MCD rats compared to controls in a previous study (Figure 1; Kim et al., 2017). Using these animals with malformed brain, we analyzed the correlation coefficient among the major structures of brain (11 ROIs) to investigate the individual changes of FC and we found abnormal functional network development in rats with MCDs. During the developmental period from P15 to P29, positive correlation of short-range FC (intra-neocortical and intra-subcortical networks) and negative correlation of long-range FC became stronger (Figures 2A–D) as shown in previous human studies (Fair et al., 2007; Dennis and Thompson, 2013). The rats with MCD showed reduced SC-brainstem connectivity compared to control rats at P15 but achieved catch-up development of SC-brainstem connectivity at P29 (Figures 2E,H, 3A).

Superior colliculus has strong descending projection to brainstem seizure circuitry, including direct projections to nucleus reticularis pontis oralis (Redgrave et al., 1987). The activation of SC exerted broad-spectrum anticonvulsant actions (Soper et al., 2016) and lesions of SC markedly attenuated seizure in genetically epilepsy-prone rats (Merrill et al., 2003). Consistent with previous studies which showed the important role of SC-brainstem circuit in generation of the spasms (Kaga et al., 1982; Chugani et al., 1992; Lado and Moshe, 2002; Pellock et al., 2010), this delayed development of SC-brainstem network in MCD rats can be associated with the spasms susceptibility in these animals (Kim et al., 2017).

In addition, the development of default mode network (DMN), aCg/pCg-Au and V1/V2/PPC-Au of MCD rats were observed only in control rats at this specific time periods (Figures 2G, 3B,C). The DMN is known as the basal network since many goal-oriented tasks deactivate this network (Raichle et al., 2001) and a similar network has also been observed in awake and anesthetized rats (Upadhyay et al., 2011; Lu et al., 2012; Schwarz et al., 2013). Many neurological and psychiatric disorders including schizophrenia (Whitfield-Gabrieli et al., 2009), Alzheimer’s disease (Greicius et al., 2004), autism (Kennedy et al., 2006) have been linked to DMN dysregulation. Thus, the developmental pattern of the DMN in control rats of this study can be considered as normal maturation of DMN and no significant changes of DMN-FC of the MCD rats can be interpreted as the delayed integration of DMN component which can be associated with the malformation of the consistent regions and the cognitive deficit and poor behavioral performances in these rats (Lucas et al., 2011; Kim et al., 2017; Hernan et al., 2018).

We also monitored direct electrophysiological signals with cortical electrodes to focus the local neuronal activities associated with this malformed cortex because RS-fMRI has the limitations of poor time-resolution and inability to detect neural electrical activities. Among the electrical activities of brain, the FO (over 30 Hz) are alleged as have a critical role in integration of neural networks during cognitive processes (Ahnaou et al., 2017; Jiruska et al., 2017) and these FO including gamma and ripples activities are associated with synchronized activation of reciprocally connected excitatory pyramidal neurons and inhibitory interneurons (Jiruska et al., 2017; Nowak et al., 2018). Moreover, the abnormal regulation of high frequency band activities were found in human patients with epileptogenic lesions (Zijlmans et al., 2012; Kim et al., 2018), schizophrenia (White and Siegel, 2016; Baradits et al., 2018), or autism (Rojas and Wilson, 2014), which suggests the FO as a biomarker of neuropsychiatric disease (Ahnaou et al., 2017).

We hypothesized that the abnormal neural network of MCD rats will lead to disrupted FO dynamics and the rats with MCD showed significant elevated gamma and ripples-ERSP at P15 and decreased ripple-ERSP at P29. The ERSP has a strong ability to detect the time-related shift of the specific band frequency (Delorme and Makeig, 2004; Makeig et al., 2004; Kim et al., 2018) and these elevated FO-ERSP of infant rats with MCD shows impaired FO dynamics in these rats. In the brains of normally developed infant rats, the density of glutamatergic synapses is too low to make physiological HFOs and epileptic HFOs can be triggered by an additional drive provided by excitatory GABA (Le Van Quyen et al., 2006). Thus, the increased FO-ERSP of rat with MCD at P15 in this study reflect the dysregulation of FO during their early development, which can be driven by abnormal excitatory GABA mediated depolarization and can be associated with the cognitive impairment and seizure susceptibility of those rats (Lucas et al., 2011; Kim et al., 2017).

On the other hands, physiological HFOs in rats appear only after the developmental GABA switch from excitation to inhibition and the formations of sufficient glutamatergic synapses after P15 (Le Van Quyen et al., 2006). Previous immunohistochemical studies with rats with exposure to MAM in utero (Lodge et al., 2009; Lewis et al., 2012; Gonzalez-Burgos et al., 2015) showed impaired neuronal network formations in these animals and the decreased ripple ERSP of rat with MCD at P29 may be the results.

Unfortunately, the direct correlation between disrupted large-scale FC and dynamic changes of local neuronal circuit could not be assessed due to the lack of the simultaneous recording of RS-fMRI and cortical EEG. However, this is the first study which identifies the change of FC and FO dynamics in young MCD rats at two different developmental periods. The rats with MCD showed delayed functional networks development and impaired cortical oscillatory dynamics during their infancy and childhood. These developmental *in vivo* imaging and EEG changes can be suggested as biomarkers for malformed brain. Further, these features can be used to get insights for the pathophysiology of cognitive dysfunction and epilepsy associated with MCD.

## Data Availability Statement

The datasets generated for this study are available on request to the corresponding authors.

## Ethics Statement

All experiments were approved by the Institutional Animal Care and Use Committee of the University of Ulsan College of Medicine and conducted in accordance with the Revised Guide for the Care and Use of Laboratory Animals.

## Author Contributions

M-JK, M-SY, W-HS, and T-SK conceived and designed the research and interpreted results of the experiments. M-JK, ML, E-JK, and YJ performed the experiments. M-JK and YJ analyzed the data. M-JK and M-SY prepared the figures and drafted the manuscript. M-SY, W-HS, and T-SK edited and revised the manuscript. All authors approved the final version of the manuscript.

## Conflict of Interest

The authors declare that the research was conducted in the absence of any commercial or financial relationships that could be construed as a potential conflict of interest.

## Footnotes

1 https://imagej.nih.gov/

2 https://www.nitrc.org/projects/mricron

3 https://afni.nimh.nih.gov/

4 https://fsl.fmrib.ox.ac.uk/fsl/FLIRT

5 https://scalablebrainatlas.incf.org/index.php
