# Supplementary figures and images for: Delayed Functional Networks Development and Altered Fast Oscillation Dynamics in a Rat Model of Cortical Malformation
Source: Front Neurosci. 2020 Aug 18;14:711. doi: 10.3389/fnins.2020.00711 (PMC7461924; doi:10.3389/fnins.2020.00711)

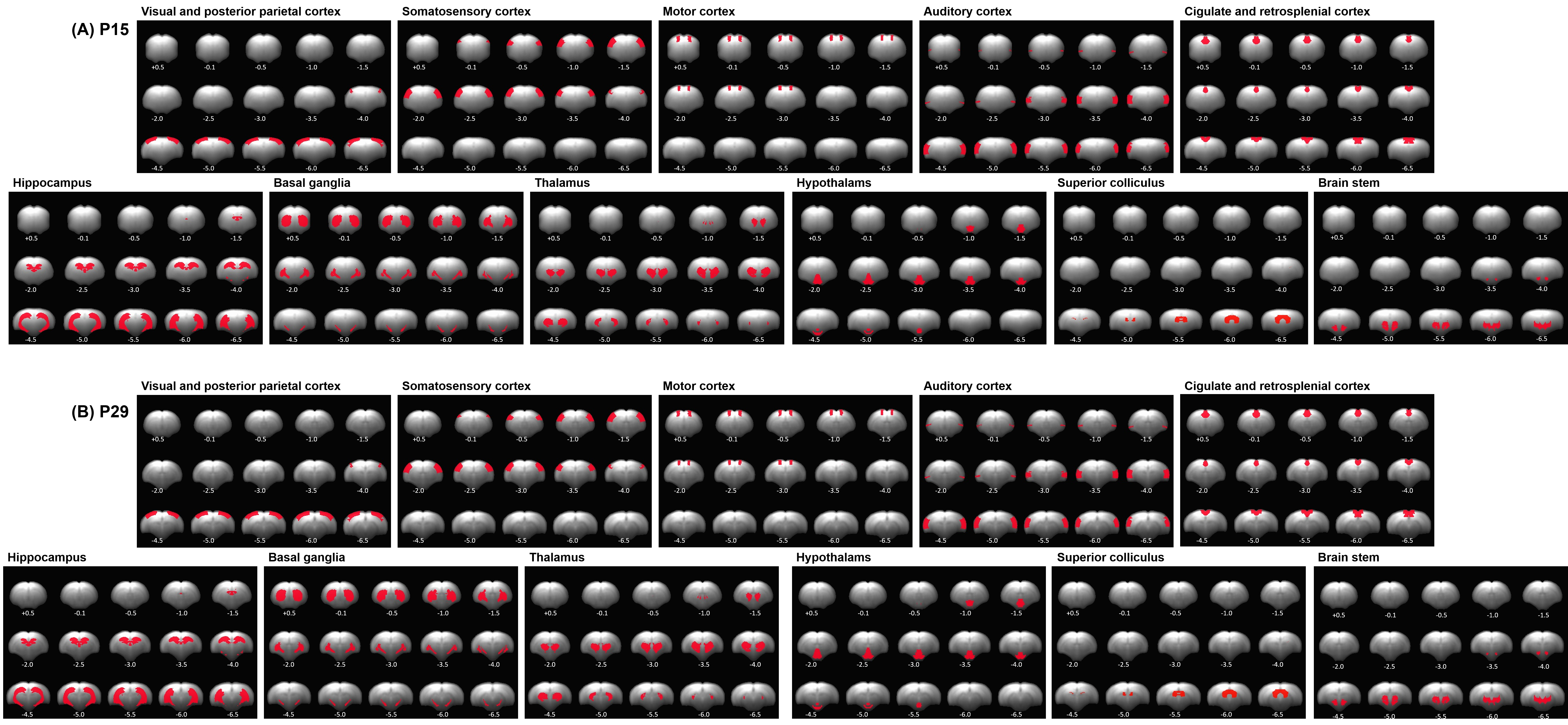

Supplement: Supplementary file 1 [file Image_1.TIF]

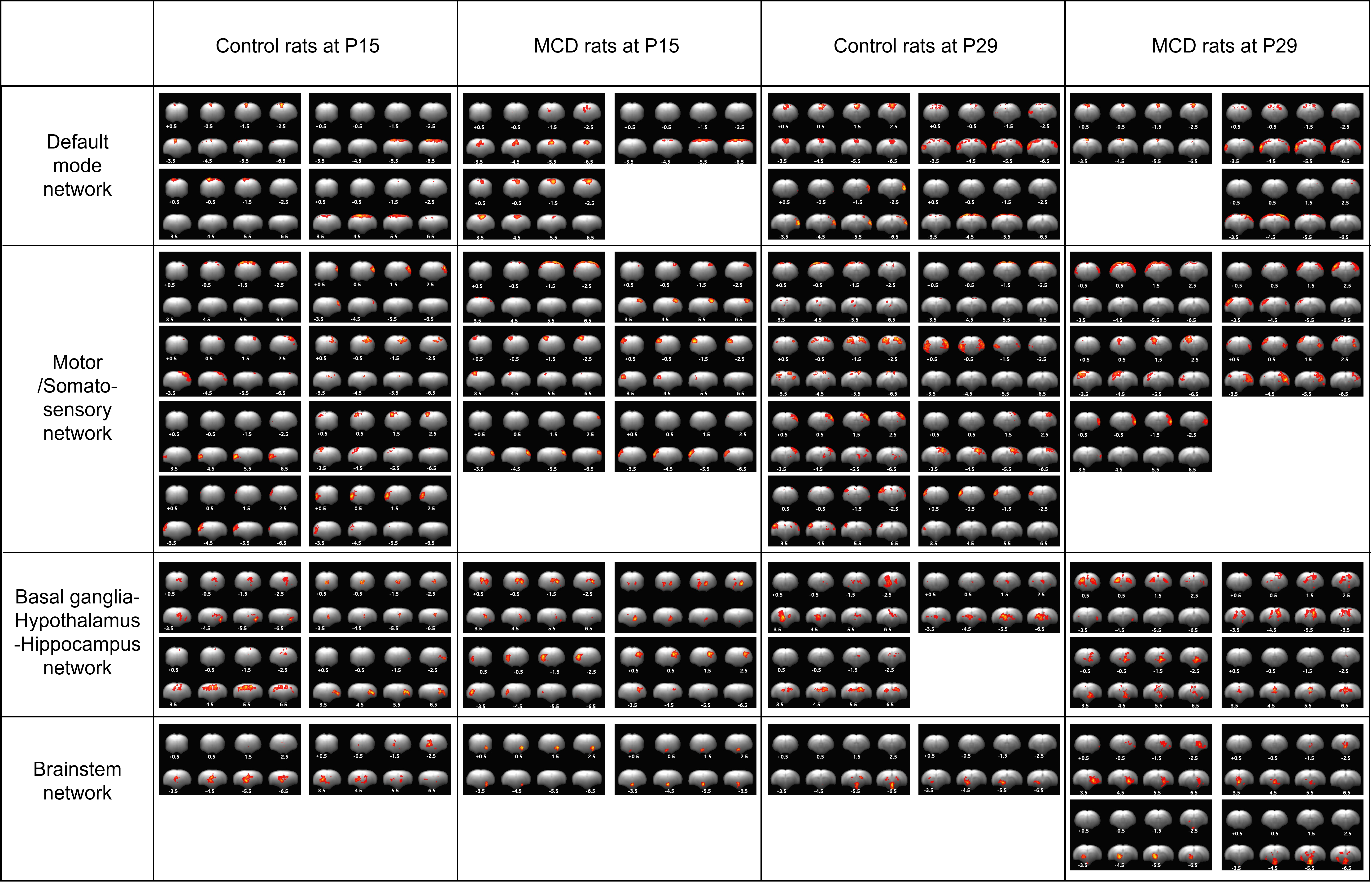

Supplement: Supplementary file 2 [file Image_2.TIF]
