# Supplementary material for: Delayed Functional Networks Development and Altered Fast Oscillation Dynamics in a Rat Model of Cortical Malformation
Source: Front Neurosci. 2020 Aug 18;14:711. doi: 10.3389/fnins.2020.00711 (PMC7461924; doi:10.3389/fnins.2020.00711)
Supplement: Supplementary file 3 [file Table_1.DOCX]

**Supplementary table 1. The rats used in these experiments**


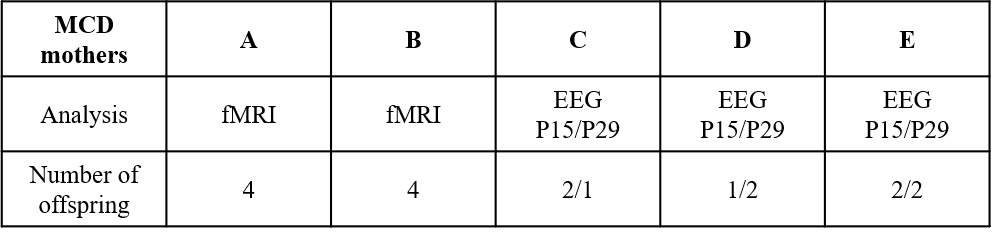

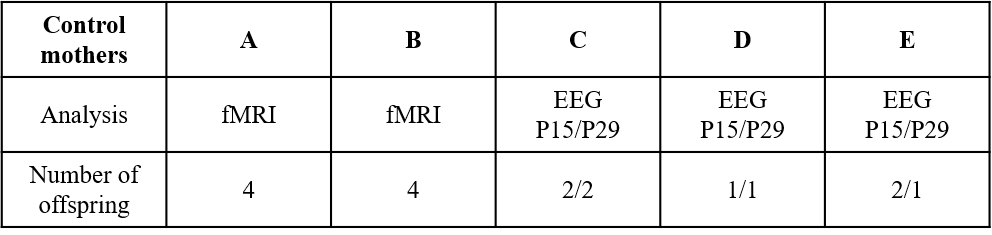


**Supplementary figure. 1. Eleven regions of interest used in rats at P15 and P29**

**Supplementary Figure 2. Spatial maps of resting-state functional networks identified using independent component analysis in control rats aged P15, rats with MCD aged P15, control rats aged P29 and rats with MCD aged P29.**
